# Mathematical biomarkers for the autonomic regulation of cardiovascular system

**DOI:** 10.3389/fphys.2013.00279

**Published:** 2013-10-07

**Authors:** Luciana A. Campos, Valter L. Pereira, Amita Muralikrishna, Sulayma Albarwani, Susana Brás, Sónia Gouveia

**Affiliations:** ^1^Center of Innovation, Technology and Education–(CITE), Camilo Castelo Branco University (UNICASTELO)Sao Jose dos Campos, Brazil; ^2^Department of Automation and Systems Technology, Federal Institute of Science and Technology of Sao PauloSao Jose dos Campos, Brazil; ^3^Department of Physiology, Sultan Qaboos UniversityMuscat, Oman; ^4^Institute of Electronics and Telematics Engineering of Aveiro, University of AveiroAveiro, Portugal

**Keywords:** heart rate variability, cardiovascular system, mathematical modeling, fuzzy logic, nonlinear dynamics, linear models, baroreflex

## Abstract

Heart rate and blood pressure are the most important vital signs in diagnosing disease. Both heart rate and blood pressure are characterized by a high degree of short term variability from moment to moment, medium term over the normal day and night as well as in the very long term over months to years. The study of new mathematical algorithms to evaluate the variability of these cardiovascular parameters has a high potential in the development of new methods for early detection of cardiovascular disease, to establish differential diagnosis with possible therapeutic consequences. The autonomic nervous system is a major player in the general adaptive reaction to stress and disease. The quantitative prediction of the autonomic interactions in multiple control loops pathways of cardiovascular system is directly applicable to clinical situations. Exploration of new multimodal analytical techniques for the variability of cardiovascular system may detect new approaches for deterministic parameter identification. A multimodal analysis of cardiovascular signals can be studied by evaluating their amplitudes, phases, time domain patterns, and sensitivity to imposed stimuli, i.e., drugs blocking the autonomic system. The causal effects, gains, and dynamic relationships may be studied through dynamical fuzzy logic models, such as the discrete-time model and discrete-event model. We expect an increase in accuracy of modeling and a better estimation of the heart rate and blood pressure time series, which could be of benefit for intelligent patient monitoring. We foresee that identifying quantitative mathematical biomarkers for autonomic nervous system will allow individual therapy adjustments to aim at the most favorable sympathetic-parasympathetic balance.

## Introduction

According to the World Health Organization (2011), hypertension and cardiovascular events continue to constitute the leading global diseases affecting more than 20% of the world's population (Harris, 2011). Although very important progress in the treatment and prevention of these diseases has been found, intense contemporary research efforts are aiming to unravel new diagnostic tools. Accumulating experimental evidence indicates that markers of autonomic nervous system such as heart rate variability (HRV) may contribute to cardiovascular diagnosis and have prognostic value (Asl et al., 2008; Ramirez-Villegas et al., 2011).

A biomarker generally refers to a key molecular or cellular event that is linked to a health outcome. Biomarkers are quantifiable and objective features of biological processes (Lee et al., 2007). Biomarkers may detect specific disease stages and processes allowing individual therapy adjustments. The development of mathematical algorithms as biomarkers to describe biological processes represents an active research area of quantitative biology and medicine. Blood pressure and heart rate are vital signs characterized by a high degree of temporal variability that is controlled by neurohumoral factors. The autonomic nervous system plays an important role in the regulation of blood pressure and heart rate through variations in its sympathetic and parasympathetic activity (Kezdi and Geller, 1968; Gootman and Cohen, 1970). Heart rate and blood pressure variability have frequently been used as biomarker of sympatho-vagal balance (Pagani et al., 1986). Sympathetic and parasympathetic systems activities are regulated through baroreflex mechanisms that tightly control blood pressure and heart rate. The baroreflex sensitivity (BRS) is a measure of baroreflex function and is defined as alterations in beat-to-beat interval (milliseconds) per unit change in blood pressure (mm Hg). BRS is influenced by various neuroendocrine systems, including central renin angiotensin system (Campos et al., 2004, 2006a) and melatonin (Campos et al., 2013b). Interplay between these systems might be responsible for the circadian alterations in cardiovascular function (Baltatu et al., 2002; Campos et al., 2006b, 2013a).

BRS may be determined pharmacologically through injection of vasoactive substances (such as phenylephrine and sodium nitroprusside) and invasive determination of arterial pressure (Campos et al., 2013b). Non-invasive methods for BRS evaluation may be through measurement of heart rate/blood pressure changes in response to deep breath, Valsalva maneuver (forced expiration against resistance), or tilt testing (passive body movement from a supine position to an upright tilt). These tests provide an index of cardiovagal function of baroreflex (Levin, 1966; Wheeler and Watkins, 1973; Borst et al., 1982; Cooke et al., 1998; Shields, 2009). However, they may be altered by some factors, including rating and depth of breathing, position of the subject, and the presence of some diseases, such as diabetic neuropathy (Sundkvist et al., 1982; Low, 1993). Continuous monitoring of heart rate offers the possibility of evaluating the spontaneous adaptations of BRS as result of dynamic variations of blood pressure (Baltatu et al., 2001; Gouveia et al., 2009). Spectral analysis of blood pressure and HRV is applied to evaluate spontaneous BRS (Head et al., 2001). Low and high frequency spectral components of the blood pressure oscillations are related with the frequency oscillations in R-R interval due to baroreflex activity (La Rovere et al., 2008). These methods for BRS evaluation have different clinical implications such as in diagnostic and clinical management of cardiovascular diseases (La Rovere et al., 2008).

### Heart rate variability

Heart rate can be defined as the number of cardiac cycles per unit of time. Cardiac cycle includes a period of relaxation (diastole) and a period of contraction (systole) of the heart, representing the time period necessary for the given event to repeat itself. The time interval between two successive R waves in the electrocardiogram (ECG) equals one cardiac cycle. Thus, the ECG can measure heart rate through the recording of electrical potentials generated by the heart's electrical activity. The frequency (*f*) is the inverse of the period (T), thus, *f* = 1/T. HRV is the variation of beat-to-beat intervals, also known as R-R intervals. The HRV indexes are obtained by analyzing the intervals between R waves, which can be captured by instruments including electrocardiograph, digital-to-analog converter and cardio frequency meter from surface electrodes that are placed at specific points on the body (Rajendra Acharya et al., 2006).

Time domain and frequency domain are two types of methods commonly used to analyze cardiovascular variability. Both methods apply to linear data structure. Time domain uses continuous monitoring of cardiovascular parameters while frequency domain uses spectral analysis to express heart rate oscillation (Task force, 1996).

### Time domain indexes of cardiovascular variability

Linear HRV analysis in time domain employs statistical methods. Data needs to be normalized before analysis. In order to make a comparison between different data sets, these have to be acquired over similar periods of time. The mostly used periods of time are 24 h (long term) and 5–30 min (short term). The time domain indexes are based on normal sinus beat-to-beat intervals (normal-to-normal, or NN), and the most commonly used are:

- standard deviation of all NN intervals (SDNN, milliseconds; Table 1). Its values depend on the length of the recording data: longer the length higher SDNN values. Therefore, a comparison of SDNN values of different length may lead to inappropriate interpretation (Task force, 1996). Low SDNN values are a predictor of high mortality in cardiovascular diseases (Kleiger et al., 1987; Task force, 1996; Nolan et al., 1998);
- root mean square of the successive differences (RMSSD, milliseconds; Table 1). It is an indication of short-term HRV components (Task force, 1996), reflects parasympathetic activity and is correlated with sudden death in epilepsy (DeGiorgio et al., 2010) and fibrillation (Dash et al., 2009).
- adjacent successive NN intervals differing more than 50 ms (NN50; Table 1) and its percentage (pNN50). It indicates short-term HRV components and reflects parasympathetic activity (Task force, 1996). The values of NN50 have been correlated with autonomic neuropathy in diabetic patients (Ewing et al., 1991).
- triangular index, calculated from the number of all NN intervals divided by the maximum of the density distribution (Table 1). Estimate the overall HRV over 24 h (Task force, 1996) and it is influenced mainly by low frequencies (Malik et al., 1989).
- triangular interpolation of NN interval histogram (TINN; Table 1). It represents the baseline width of the distribution measured as a base of a histogram triangle approximating the RR interval distribution (Malik and Camm, 1993; Vanderlei et al., 2009). Estimate overall HRV over 24 h (Task force, 1996) and it is influenced mainly by low frequencies (Malik et al., 1989).

**Table 1:**
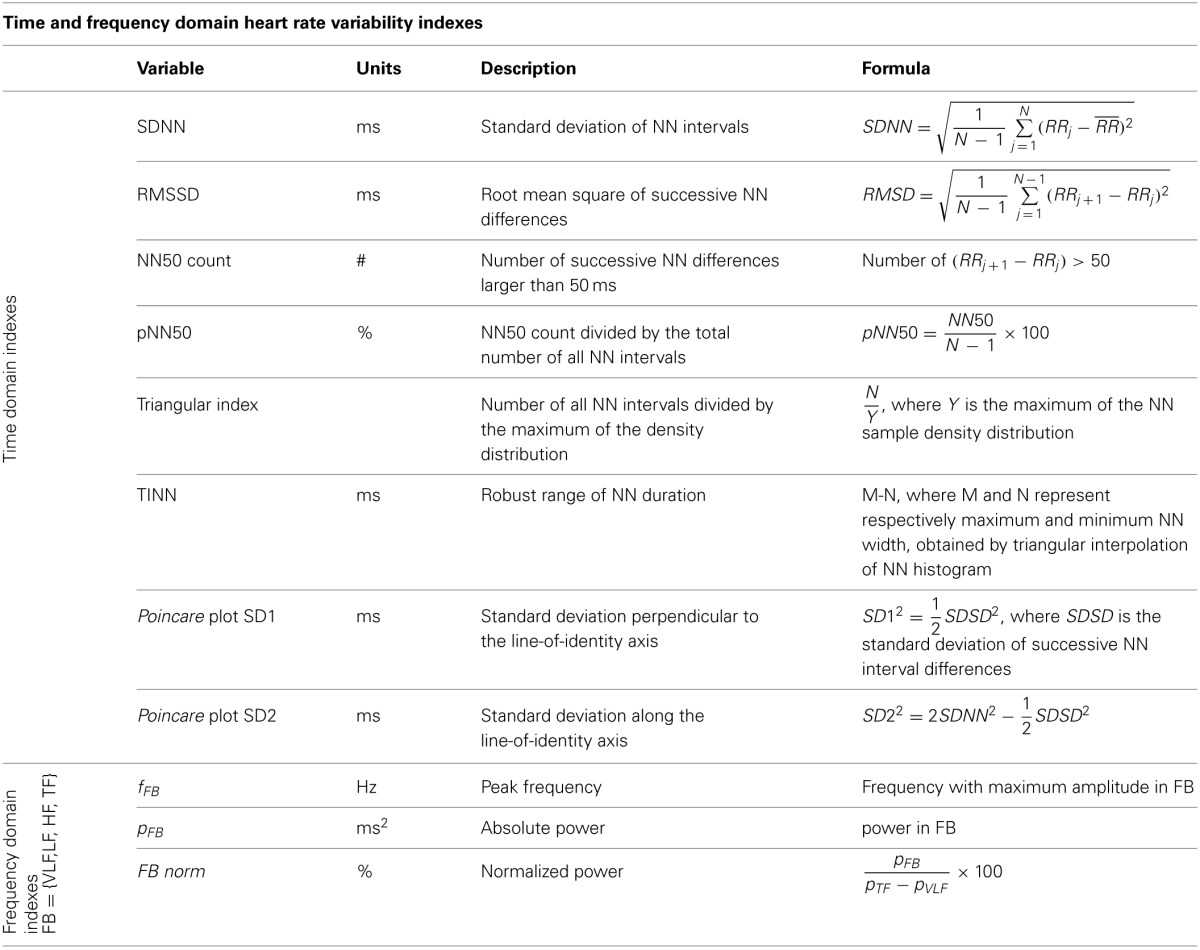
**Time and frequency domain measures of heart rate variability (Kamen and Tonkin, 1995; Task force, 1996; Brennan et al., 2001)**.

FB, frequency bands; VLF, very low frequency; LF, low frequency; HF, high frequency; TF, total frequency.

### Frequency domain indexes of cardiovascular variability

Linear HRV analysis in frequency domain employs mathematical algorithms for frequency assignment. Physiological data collected as a time series can be considered as a sum of sinusoidal oscillations with different frequencies. The conversion of time domain analysis to frequency domain can be done through a mathematical transformation developed nearly two centuries ago (1807) by a French mathematician named Jean Baptiste Joseph Fourier (1768–1830). This process, called spectral analysis, allows signal decomposition originated from time series (*tachogram*) in its different frequency components, or in the so-called frequency bands. Noteworthy is that frequency refers to the number of times that a particular phenomenon occurs related to time. Typically, the unit for frequency is Hertz (Hz), which is equivalent to one cycle per second.

From continuous recordings of heart rate, the total power (TP) is decomposed into three distinct bands in humans:

1. high frequency band (HF; Table 1) with frequency ranging from 0.15 to 0.40 Hz, related to the heart rate variations associated with the respiratory cycle, commonly called respiratory sinus arrhythmia. It is usually modulated by the parasympathetic nervous system (Kuusela et al., 2003; Rajendra Acharya et al., 2006). HF is also known as “respiratory” band because it corresponds to the R-R fluctuations caused by breathing (Bernardi et al., 1989; Ori et al., 1992; Bernardi et al., 1995);
2. low frequency band (LF; Table 1) with frequency ranging from 0.04 to 0.15 Hz is modulated by both sympathetic and parasympathetic nervous systems (Kuusela et al., 2003; Rajendra Acharya et al., 2006);
3. very low frequency band (VLF; Table 1) with frequency ranging from 0.003 to 0.04 Hz, it is a variable which depends on the renin-angiotensin system, whose regulation is also affected by sympathetic and parasympathetic nervous systems (Taylor et al., 1998; Kuusela et al., 2003; Acharya et al., 2004). The lower frequency of the VLF band depends on window length. It was demonstrated an association of VLF with mortality after cardiac infarction (Bigger et al., 1992).

In the analysis of fetal heart rate (FHR) tracings, these frequency bands are usually adjusted to VLF (0–0.03 Hz), LF (0.03–0.15 Hz), and HF (0.5–1 Hz), and hold the same physiological associations as in human adults. FHR recordings additionally exhibit a MF frequency band related to the fetal movements and maternal breathing (Signorini et al., 2003).

These frequency bands are not applicable for smaller animals such as rats and mice. In rats, the components of VLF to HF of HRV power spectra vary between 0.0 and 3.0 Hz (Task force, 1996; Baltatu et al., 2001; Bezerra et al., 2001; Ushizima et al., 2001; Silva et al., 2009), while in mice, the components of LF to HF vary between 0.1 and 5.0 Hz (Ishii et al., 1996; Joaquim et al., 2004; Baudrie et al., 2007).

Measurement of spectral components is typically made in absolute power (ms^2^). However, the values of LF and HF can also be expressed in normalized units (NU) representing the value of each component relative to the TP minus the VLF component (Task force, 1996).

Both time and frequency domain methods for variability evaluation analyze linear properties of the data, as described above. These methods have limitations, as they require windowing of the data and it uses deterministic algorithms that are valid only to periodic phenomena. Therefore, there is a need to identify a hierarchy of models, each suited for a different type of investigation or to different parts of the system and devise strategies to couple them, using a multiscale framework. Systems of differential-algebraic equations can be employed to study systemic behavior and auto-regulation mechanisms.

Heart period and blood pressure are coupled in a closed loop. The coupling between heart period and blood pressure via baroreflex regulation can be evaluated using cross-correlation analysis (Silvani et al., 2008, 2011), regression analysis by the events technique (Gouveia et al., 2009) and causal estimates (Acharya et al., 2004; Porta et al., 2011). Cross-correlation analysis gives a correlation coefficient between variations of heart period and blood pressure (Silvani et al., 2011). Geoffrey Head et al. demonstrated that cross-spectral coherence transfer function between heart period and blood pressure could be used effectively to reproducibly estimate BRS (Head et al., 2001). The transfer function gives information about coherence, gain and phase relation of both signals (Baltatu et al., 2001). Coupling between heart period and systolic blood pressure in healthy subjects varies among wake-sleep states (Silvani et al., 2008). It was suggested that cross-correlation function is applicable to study the balance between central autonomic and baroreflex control of heart rate. For instance, there are positive and negative correlations between heart period and blood pressure values that result from baroreflex and central autonomic controls, respectively (Silvani et al., 2011). However, in cases of BRS impairment, cross-correlation is likely to be statistically not significant, due to autonomic balance alterations. To address this shortcoming, the so called events technique based on baroreflex events, was proposed to maximize simultaneously the number of beats considered for BRS estimation and the correlation between the corresponding systolic blood pressure and RR values, in order to provide a more accurate baroreflex slope estimate (Gouveia et al., 2009). This technique has shown to accurately detect baroreflex alterations, associated with postural changes (Gouveia et al., 2009) and drug-induced BRS stimulation (Beloka et al., 2009). This technique has also been demonstrated to be capable of providing segments of short and of long beat length, which were associated with parasympathetic and the sympathetic ANS activities, respectively (Gouveia et al., 2010).

One of the disadvantages is that cross-correlation and linear regression methods are able to estimate only the linear component of heart period and systolic blood pressure coupling (Silvani et al., 2008; Gouveia et al., 2009). The causal relationship theory between heart period and systolic blood pressure was introduced by Oppenheim (Oppenheim and Schafer, 1975). This led to studies to find the dominant causal direction in the interactions between heart period and systolic blood pressure. Cross-conditional entropy was used to study heart pulse-systolic blood pressure causality during head-up tilt test in heart transplanted patients, and compared with classical approach of linear methods. The head-up tilt induced progressive shift from the prevalent causal direction to the reverse causality in healthy subjects (Porta et al., 2011). Although cross-conditional entropy is a tool to determine the causality, it is unable to account for the combined exogenous influences of respiration on R-R interval and systolic blood pressure variability (Baselli et al., 1994; Cooke et al., 1999; Westerhof et al., 2006; Porta et al., 2011).

### Non-linear indexes of cardiovascular variability

The nonlinear theory has been growing among physiologists and physicians aiming to explain the workings of biological phenomena, highly complex, dynamic, and interdependent, where the system behavior differs from the behavior of its parts or elements (Huikuri et al., 2003).

The exponent of power-law, approximate entropy (ApEn) analysis and detrended fluctuation (DFA) are nonlinear methods recently introduced to the study of HRV.

Entropy is a measure of randomness or disorder, as included in the second law of thermodynamics, namely the entropy of a system that tends toward the maximum. Different states of a system tend to evolve from ordered configurations to less organized settings, but statistically more likely. Referring to the time series analysis, the ApEn provides a measure of the degree of irregularity or randomness within a series of data. Entropy was originally used by Pincus (1991) as a measure of system complexity, where smaller values indicate greater regularity, and higher values lead to more disorder, randomness, and complexity of the system. For instance with a drop in the ApEn, heart rate becomes more regular with age in both men and women (Ryan et al., 1994).

The DFA is a technique that characterizes the variation pattern through measuring scales. DFA has been specifically developed to distinguish between intrinsic fluctuations generated by the complex system and those caused by external or environmental stimuli acting on the system (Peng et al., 1995). The variations that arise due to extrinsic stimulation are presumed to cause a local effect, while the intrinsic variations due to the dynamics of the system are assumed to exhibit a long-term correlation.

The analysis of the *Poincare* plot or Lorenz plot is considered as based on nonlinear dynamics by some authors (Kamen and Tonkin, 1995; Voss et al., 2007; Vanderlei et al., 2010). The *Poincare* plot is a two-dimensional graphical representation of the correlation between consecutive RR intervals, where each interval is plotted against the next one (Lerma et al., 2003), and its analysis can be done qualitatively (visually) by evaluating the shape formed by its attractor, which shows the degree of complexity of the RR intervals (Woo et al., 1992), or quantitatively, by fitting an ellipse to the figure formed by the plot from where the indexes are taken: SD1, SD2, and SD1/SD2 ratio (Tulppo et al., 1996; Vanderlei et al., 2010). SD1 represents the dispersion of points perpendicular to the line of identity and appears to be an index of instantaneous beat-to-beat variability (i.e., the short-term variability which is mainly caused *by respiratory sinus arrhythmia*), while the SD2 represents the dispersion of points along the line of identity and it characterizes long-term HRV. The SD1/SD2 ratio shows the relationship between short and long-term RR interval variations (Gamelin et al., 2006; Rajendra Acharya et al., 2006). Despite the fact that Poincaré plot is primarily considered a nonlinear technique, it has been shown that SD1 and SD2 can be obtained as a combination of linear time domain HRV indexes (Brennan et al., 2001, Table 1). Therefore, alternative measures are still needed to characterize nonlinear features in Poincaré plot geometry.

### Fuzzy logic concepts

The possibility of using mathematical methods and theories for data analysis has opened up a range of possibilities for the study of pathophysiological behaviors of cardiovascular variability (Hu et al., 2009; Sassi et al., 2009; Gieraltowski et al., 2013). Large volume of data can be more easily assessed and analyzed with fuzzy logic. In order to better understand the onset and development of important pathologies, the autonomic nervous system activity can be explored through dynamical fuzzy logic models, such as the discrete-time model and the discrete-event model. Fuzzy logic approaches are able to perform non-linear mapping or predictions involving more than one cardiovascular parameter and to explore possible relations among these parameters, which normally would not be considered as a possibility. Fuzzy logic represents a flexible system that adequately describes nonlinear and complex systems since the resulting function can be written as a weighted linear combination of the system inputs and, therefore, it can resemble a nonlinear function as needed. For this reason, fuzzy logic methods are a feasible solution to consider in the absence of prior mathematical description between input-output variables (Kovacic and Bogdan, 2006).

Considering the Sugeno Fuzzy Logic formulation, the system output z can be modeled from

$$z=\frac{\sum_{i\;=\;1}^{N}w_{i}z_{i}}{\sum_{i\;=\;1}^{N}w_{i}},$$

where *N* corresponds to the number of fuzzy rules and $z_{i}=\sum_{j\;=\;1}^{n}a_{i}x_{j}+c_{i}$ is a linear combination of the system inputs *x*_j_, *j* = 1, … n. The rule weights are obtained as $w_{i}=\prod_{j\;=\;1}^{n}\Gamma_{F_{j}^{i}}(x_{j})$ where Γ _*F*^*i*^_*j*__ is the membership function of rule *i* and input *x*_j_. Although membership functions may assume different shapes, the Gaussian function is rather a popular choice in the literature due to its symmetry and dependence on mean and variance, which correspond respectively to the center and the width of the membership function.

Fuzzy logic has the singular characteristic to combine empirical knowledge (described as linguistic rules) and knowledge directly extracted from the data (Sadegh-Zadeh, 1999), enabling an easier way to interpret the outcomes in a physiological perspective. This mathematical model may be a reliable method to evaluate the influence of the autonomic nervous system over cardiovascular control in healthy and diseased subjects (Carvalho et al., 2002).

The main advantage of the use of fuzzy logic systems comes from their power to deal adequately with the uncertainty (Zadeh, 1975; Kovacic and Bogdan, 2006). In particular, this approach tolerates imprecise data, and it is focused on the “plausibility” of occurrence rather than the traditional binary response “0” or “1.” For example, while a given measurement of a certain biological variable such as stress may convey a person as being “content,” the same measurement may reveal a status of “dissatisfaction” for another one. Thus, biological variables that vary from person to person and are closely influenced by external and internal changes direct themselves toward fuzzy logic model of analysis, where the application of methods of investigation based on zero and one, true and false does not apply (Zadeh, 1975). Cardiovascular signals are characterized by a great intra- and inter-individual variability, besides imprecise measurements due to limited resolution of acquisition systems. Additionally, it is believed that traditional statistical methods may not capture all the information needed to describe disease in its complexity and dynamics (Grossi, 2005). In this context, fuzzy logic may be a more reliable alternative to traditional methods.

### Applications of fuzzy logics to the analysis of cardiovascular variability

Fuzzy logic approaches have been recently used in the cardiovascular field in different contexts including applications in signal processing and monitoring, classification, prediction or control. One approach consists of extracting the relevant features from one or more cardiovascular signals, which are then integrated into a fuzzy logic scheme aiming at the identification of the presence or the quantification of a pathological state.

Fuzzy logic methods have been successfully integrated in control systems. For instance during anesthesia, mean arterial pressure was controlled based on the error between desired and measured values, allowing it to control the balance between the unconsciousness and the side effects caused by the hypnotic drug (Meier et al., 1992). Also during anesthesia, hemodynamic changes were successfully modeled considering drug dose level alterations as inputs of the fuzzy system (Nunes and Amorim, 2008). In hemodialysis condition, fuzzy logic has also shown to be capable of effectively control blood pressure trends, using ultra-filtration rate as input (Mancini et al., 2007). Such a system allowed an overall reduction of 40% of the most severe episodes in hypotension-prone subjects.

Abnormal cardiac rhythms have been identified using artificial neural network and fuzzy interactions based on nonlinear heart period R-R features, such as spectral entropy, *Poincare* SD1/SD2, and Lyapunov exponent (Acharya et al., 2004). Also based on R-R features, fuzzy logic was used for ECG beat classification to detect arrhythmic and ischemic heartbeats (Tsipouras et al., 2007). Fuzzy logic approaches showed efficiency in improving oscillometric cuff pressure measurements by properly detecting outliers and noise artifacts (Lin et al., 2003).

With the goal of evaluating autonomic nervous system function, fuzzy logic has been used to choose the optimum subset of time, frequency and nonlinear variables related to sympathetic and parasympathetic activities on HRV (Petkovic et al., 2013). Fuzzy logic approach has been used in a classification scheme to jointly evaluate results of several autonomic tests, *e.g.,* head-up tilt test and active postural change, using both time and spectral analysis of heart rate and of diastolic blood pressure series (Carvalho et al., 2002). Similar fuzzy logic schemes were used for the information fusion of relevant features extracted from multimodal cardiovascular signals, such as heart period R-R and systolic blood pressure, for the detection of life threatening states in cardiac care units (Kannathal et al., 2006).

Recently, fuzzy logic methods have been employed to effectively describe blood pressure and heart period R-R coupling and, therefore, have the potential to improve time domain BRS estimation (Liu et al., 2008; Gouveia and Bras, 2012). The autoregressive linear analysis approach for BRS estimation has limitations when cardiovascular regulation is depressed. Liu et al. proposed a hybrid model consisting of a parallel modular structure with an autoregressive and a fuzzy logic system, to study simultaneously linear and non-linear heart rate and blood pressure coupling mechanisms (Liu et al., 2008). This approach illustrates the utility of combining more traditional methods with fuzzy logic, which could be of advantage in diseased conditions when cardiovascular system regulation is afflicted.

**Figure 1:**
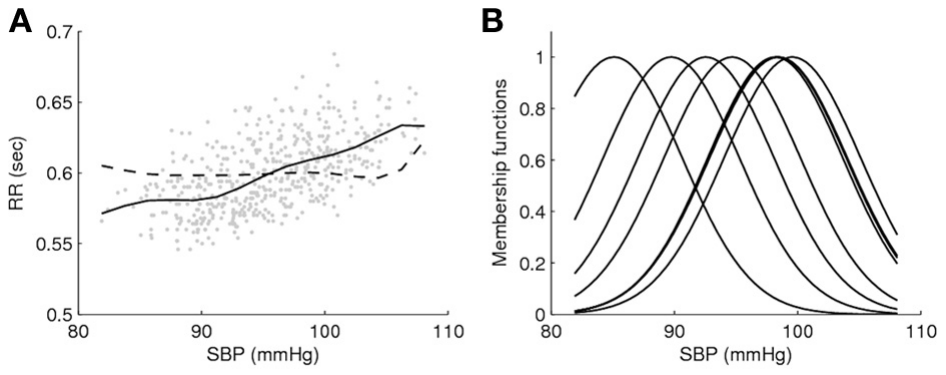
**Fuzzy functions obtained for one healthy subject: (A) Fuzzy curve in solid line was obtained from real data (represented in gray) and Fuzzy curve in dashed line was estimated from an isodistribution surrogate realization; (B) membership functions obtained from real data**.

**Figure 2:**
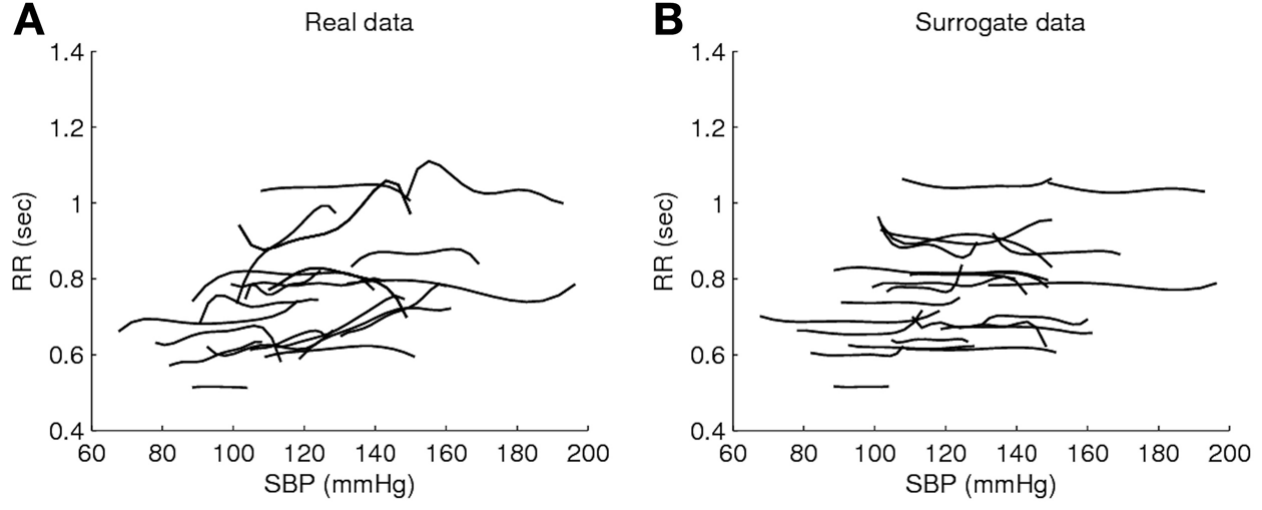
**Fuzzy surfaces obtained for a set of 23 subjects in Lying condition [EuroBaVar dataset (Gouveia and Bras, 2012)], with a curve in each subplot according to one subject.** Subplot **(A)** shows the fuzzy curves obtained from real data and subplot **(B)** shows the fuzzy curves estimated from an isodistribution surrogate data.

Time domain BRS methods based on spontaneous data typically assume blood pressure and heart period R-R linearity and provide single slope estimation, regardless of the blood pressure value (Beloka et al., 2009; Gouveia et al., 2009). In this context, fuzzy logic methods can contribute to establish a BRS dependent of blood pressure level, similarly to time domain blood pressure pharmacological methods. Recently, fuzzy logic has been used to analyze spontaneous R-R series as a function of blood pressure values, comparing performances in real and surrogate data (Gouveia and Bras, 2012). As an illustrative example, Figure 1 shows fuzzy logic curves and the membership functions for a healthy subject. The fuzzy logic curve obtained from real data is much less flatter than that obtained from isodistribution surrogate data (gained by shuffling the RR data) and exhibits significantly lower average modeling errors (Figure 1A). Also, the non-uniform location of the membership functions suggests that different systolic blood pressure values contribute differently to RR modeling (Figure 1B). These results indicate that fuzzy logic is able to model R-R changes connected to blood pressure alterations, besides the mean value and, thus, has the potential to improve time domain BRS estimation in spontaneous conditions. It remains to be assessed the clinical impact of these findings and inherent repercussion on BRS estimation. Finally, Figure 2 illustrates the flexibility of fuzzy logic-based modeling in the identification of heterogeneous shapes and patterns in systolic blood pressure and RR association for a set of several subjects.

Given the complexity of the mechanisms regulating heart rate and its non-linear characteristics, it is reasonable to assume that HRV analysis based on non-linear methods would generate valuable knowledge on the systems involved in the HRV regulation. The mainly used non-linear method of HRV analysis is *Poincaré* plot, which although simple and easy to use, it does not reflect the number of samples at each point of the graph, leading to errors of judgment of its plots (Hnatkova et al., 1995). The use of fuzzy logic and the consequent use of a “plausibility” rather than a binary logic may help to overcome the drawbacks found in the traditional methods of analysis.

## Concluding remarks

Fuzzy logic is a suitable choice when the system deals with uncertainty data, when linguistic interpretation is needed and when data “plausibility” should be taken into account. Fuzzy logic has been successfully used in different scenarios for the analysis of cardiovascular variability, with special emphasis on control and classification systems. Recent studies point out fuzzy logic also as a modeling alternative for cardiovascular time series, with potential impact on the estimation of joint parameters, e.g., arterial BRS. Therefore, fuzzy logic is a promising approach for the analysis of cardiovascular system and its regulatory mechanisms in normal and diseased conditions.

We expect an increase in accuracy of modeling and a better estimation of the heart rate and blood pressure time series, which could be of benefit for intelligent patient monitoring. We foresee that identifying quantitative mathematical biomarkers for autonomic nervous system will allow individual therapy adjustments to aim at the most favorable sympathetic-parasympathetic balance.

### Conflict of interest statement

The authors declare that the research was conducted in the absence of any commercial or financial relationships that could be construed as a potential conflict of interest.
